# Population structure of yellowtail snapper using age-based life history and otolith shape in southern Gulf of México

**DOI:** 10.1371/journal.pone.0320012

**Published:** 2025-04-16

**Authors:** Ximena Renán, Gabriela Galindo-Cortes, Isabel Cervantes-Camacho, Mariana Ramírez, Marco A. Pasos, Teresa Colás-Marrufo, Thierry Brulé

**Affiliations:** 1 Centro de Investigación y de Estudios Avanzados del Instituto Politécnico Nacional, Departamento de Recursos del Mar, Mérida, Yucatán, México; 2 Instituto de Ciencias Marinas y Pesquerías, Universidad Veracruzana. Boca del Río Veracruz, México; University of Messina, ITALY

## Abstract

Yellowtail snapper *Ocyurus chrysurus* is commercially important throughout its distribution range. In southern Gulf of México, it is caught as part of a largely undocumented multispecific snapper fishery. Samplings were done at three fishing sites with the highest landings volume of Yucatán, México: Celestún, Dzilam de Bravo, and Río Lagartos. Age and growth parameters were generated with the von Bertalanffy growth model via *annuli* counts in otolith thin sections of 1,124 individuals. Marginal increment analysis confirmed that opaque zone formation is annual and occurs from July to September. Overall age range was 0^ +^ to 12 years (17.7 to 38.9 cm fork length) but differed between Celestún (0^ +^ to 9 years), Dzilam de Bravo (1 to 12 years), and Río Lagartos (2 to 12 years). Growth parameters and lifespan for combined sexes also varied between sites: Celestún (L_ ∞_ =  41.59 cm, K =  0.11 year^-1^, *t*_*max*_ =  22.41 years); Dzilam de Bravo (L_ ∞_ =  38.36 cm, K =  0.16 year^-1^, *t*_*max*_ = 15.5 years); and Río Lagartos (L_ ∞_ =  40.28 cm, K =  0.12 year^-1^, *t*_*max*_ =  20.01 years). Age at maturity was 1.3 years for females and < 1 year for males. Natural mortality based on *t*_*max*_ was 0.28 year^-1^ overall. A principal coordinates analysis identified morphometric variables as explaining 92.2% of otolith differentiation by fishing site, with average centroid distances: Celestún, 1.98; Dzilam de Bravo, 2.22; and Río Lagartos, 2.75. Differences in growth rate, lifespan, natural mortality and otolith shape indicate that *O. chrysurus* in southern GoM exhibits structural complexities that suggest the occurrence of repercussions from fishing-induced demographic changes.

## Introduction

Yellowtail snapper *Ocyurus chrysurus* (Bloch 1791) is a demersal species found in western Atlantic Ocean from Massachusetts to southern Brazil, including the Bahamas, southern Florida, Caribbean Sea and Gulf of México [1]. Juveniles inhabit areas with seagrass beds and mangroves in brackish or marine waters at shallow depths. Adults inhabit reefs with hard and rocky bottoms, at depths from -20 to -40 m, without evident bathymetric distribution [2]. Individuals make ontogenetic migrations between settlement sites, juvenile areas and adult areas where spawning aggregations occur [2]. *Ocyurus chrysurus* is a carnivorous and opportunistic generalist species that mainly consumes benthic prey such as crustaceans and fish [3]. In the southern Gulf of México (GoM), it has a long reproductive season with a spawning peak from March to June. Females reach sexual maturity at 21.3 cm fork length (FL) and males at 19.4 cm (FL) [4].

No specific catch volume records are kept for the *O. chrysurus* fishery in México. Catches of *Ocyurus chrysurus* and *Lutjanus synagris* (Linnaeus 1758, Lane snapper) are labelled under a single “snapper” category while *Lutjanus campechanus* (Poey 1860, Northern red snapper) catches are reported separately [5]. *Ocyurus chrysurus, L. synagris* and *L. campechanus* are the main targets in the snapper fishery of the southern GoM, contributing with 85% of the annual landings volume and are the main export species to US markets [6]. Despite their commercial importance, the only direct regulatory instrument applied in México’s snapper fisheries is controlled access through a commercial fishing permit; and indirectly, as bycatch species in the *Epinephelus morio* (Valenciennes 1828, Red grouper) fishery (NOM 065 SAG/PESC-2014), in which permitted fishing gear is specified [7,8]. The GoM snapper fishery account for 30% of the total national snapper catch; the State of Yucatán produced an average of 580 metric tons per year in the five years prior to 2022 [9]. *Ocyurus chrysurus* landings of the Yucatán artisanal fleet have steadily increased in average yield (kg/fishing trip), and it has become the most abundant species in landings on the state’s west (Celestún) and northeast (Río Lagartos) coasts, even surpassing *E. morio*, the primary target species in the state’s fin-fish fisheries [8]. Before 2018, *O. chrysurus* fishery was classified as healthy, but now, the southern GoM stock is considered as experiencing overfishing, with increasing exploitation [6].

Information on stock complexity is essential for understanding stock dynamics and the differential response of a population or subpopulation to anthropogenic and environmental pressures [10,11]. Different approaches are used to describe fish population structure since identification of management units and their spatial patterns are the basis for developing management and conservation strategies focused on sustainable exploitation. Analysis of individual growth and otolith morphometry are used to describe population structure linked to exogenous and endogenous variables [12]. Fish growth rates exhibit wide intraspecific ranges under different environmental factors such as salinity, water temperature or depth, and feeding conditions, due to the energy and nutrient supply available to the individual [13]. On the other hand, individuals inhabiting different habitats have unique, spatially identifiable otolithic chemical and shape signatures reflecting the characteristics of the environment and the time the fish occupied a certain area, which can be used to discriminate their stocks [14,15].

The description of population structure based on age and growth for *Ocyurus chrysurus* is imperative for the quantification of the population’s productivity. The following biological characteristics of the species are expected to be useful for the description of demographic changes: Firstly, the species exhibits a high degree of fidelity to the sites it inhabits [2]. Secondly, its feeding habits are diverse, with changes in diet as it grows [3]. Thirdly, the species is subject to unregulated fishing pressure [8]. And finally, the three sites under analysis are the Yucatán sites with the highest species’ landings and differ in environmental characteristics. The acquisition of *O. chrysurus* population structure in the southern GoM, using age-based life history (age, growth rate, lifespan, natural mortality and age at maturity) and otolith morphometry, will facilitate the comprehension of the species’ responses to potential negative repercussions caused by fishing, such as a progressively younger reproductive population and decreased productivity. Consequently, the vital life history parameters and demographic aspects characterized will be useful in stock assessments and management of the species.

## Methods

### Sampling and data collection

**Fig 1:**
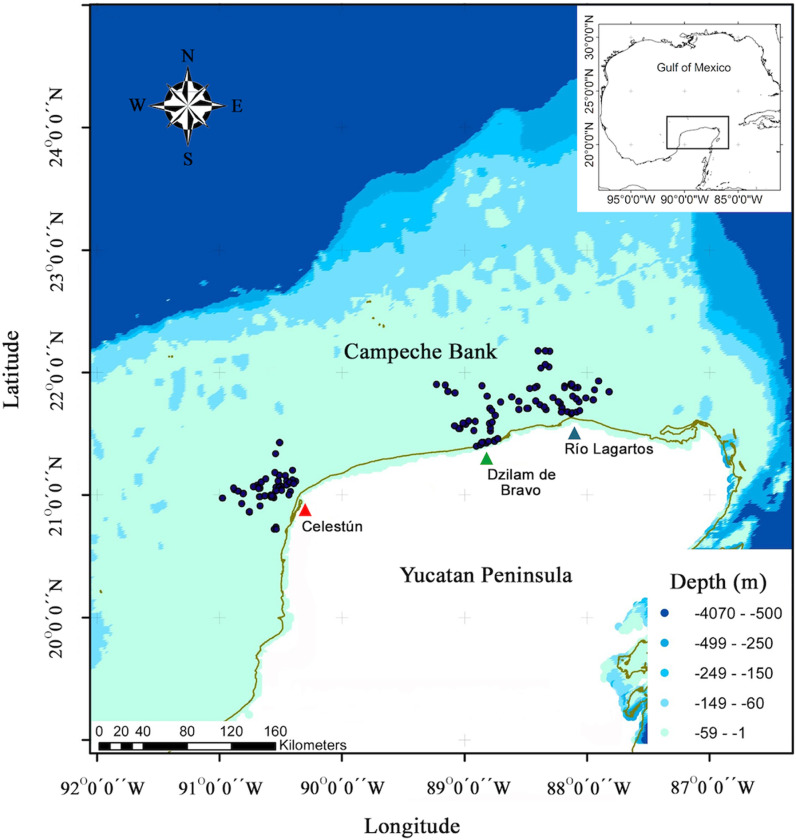
*Ocyurus chrysurus* sampling sites during 2008 – 2009 in southern Gulf of México. Triangle markers showed the three artisanal-fishing ports with the highest catches volume.

The study area consists of ocean waters near the fishing ports of Celestún, Dzilam de Bravo and Río Lagartos in the southern GoM on the continental shelf of the Yucatán Peninsula (19 – 24 °N, 93 – 86 °W; Fig 1). The waters off the northeast coast of Yucatán, near the port of Río Lagartos, are influenced by the Yucatán Current, which flows from the Caribbean Sea into the southern GoM (Loop Current), and during the summer, by an upwelling of enriched cold waters (16.0 – 20.5 °C) [16]. On the central and western coasts (near Dzilam de Bravo and Celestún, respectively) marine waters mix with upwellings of subterranean freshwater from fractures in the karst system [17,18]. The three sites have different biotopes as well. Río Lagartos has high topographic complexity with sandy and hard bottoms covered by calcareous algae, hard corals and octocorals. Dzilam de Bravo has seafloor covered mainly by seagrass beds, algae and small semi-circular sponges. Celestún has hard seafloor, with slabs, stones and extensive sandbanks harboring some algae, hard corals and abundant gorgonians [19].

A total of 1,449 *O. chrysurus* individuals were collected. They were caught in monthly samplings during 2008 – 2009 at depths between -2.5 and -25 m by artisanal fishers using handlines and hooks. No bathymetric distribution has been reported for the species, but some of the largest individuals might be outside the reach of the artisanal fleet. Sampling sites were located in the waters off the three mentioned fishing sites: Celestún, Dzilam de Bravo, and Río Lagartos (Fig 1), georeferencing the position of the catch. Once in the laboratory, data collected for each individual were: total length (TL), fork length (FL), and standard length (SL), all in centimeters (cm), as well as whole-body weight (WW) and gutted weight (GW), in grams (g). Each pair of sagittal otoliths was extracted through the gill arch, cleaned with 70% alcohol, and weighed in pairs using an analytical balance (precision: 0.1 mg); average otolith weight (OW) was calculated from these data. Age of maturity was obtained using individual sex and maturity condition data from previous gonad histological analysis results for the same individuals [4].

The specimens analyzed were deceased organisms collected from the artisanal fishing fleet operating in the waters of Yucatán at southern GoM. No live animals were subjected to experimentation, and the biological samples obtained from each deceased specimen were handled with the utmost care to prevent any impact on their subsequent commercialization. In line with ethical considerations, fishermen are required to adhere to the Official Mexican Wildlife General Law (Ley General de Vida Silvestre), ensuring that animals were treated with dignity and respect by minimizing pain, physical harm, and suffering during capture and commercialization [20].

### Age and growth

**Fig 2:**
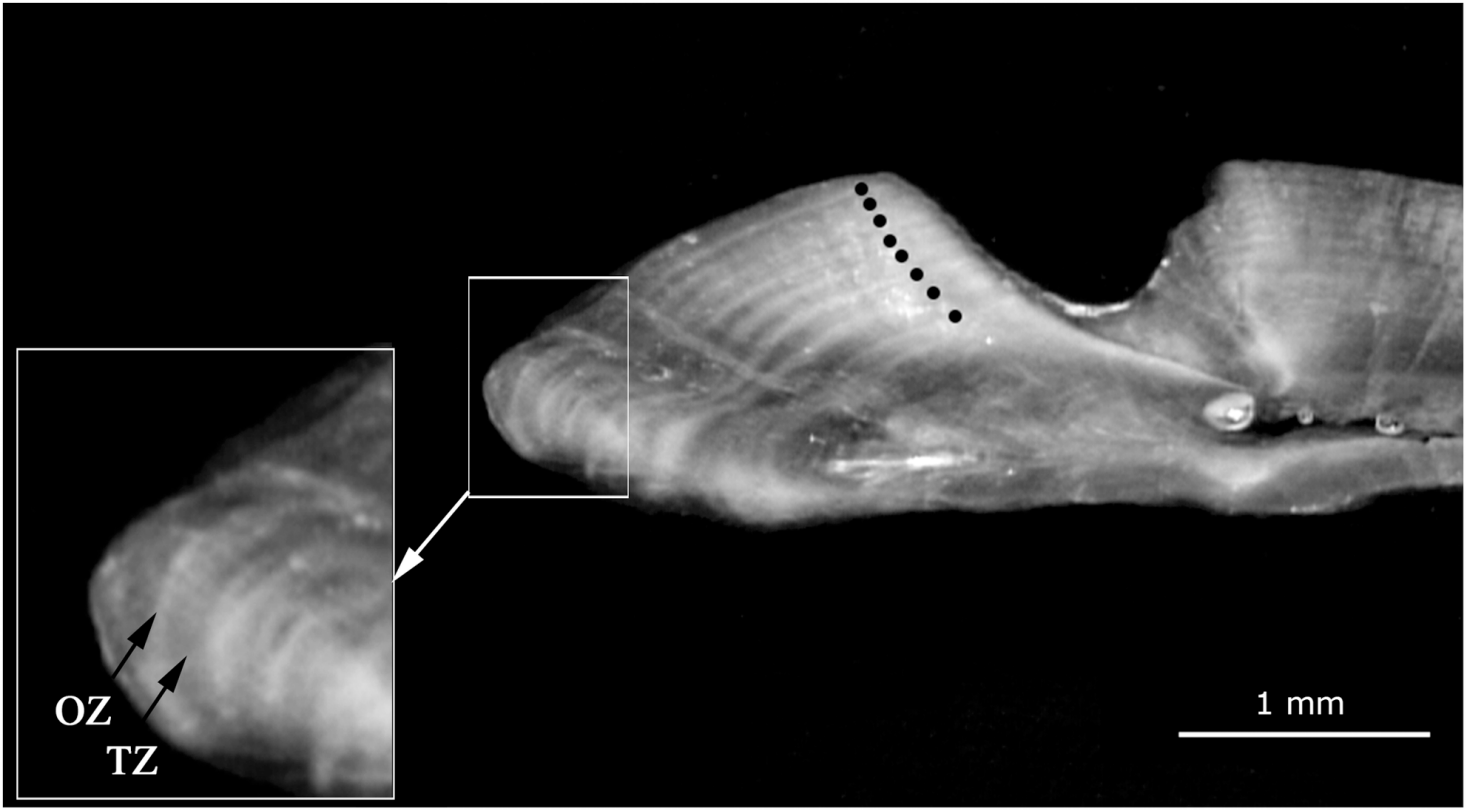
*Ocyurus chrysurus* otolith thin section. *Annuli* in dark circles, represent the individual´s age in years. Bright opaque zone (OZ) and dark translucent zone (TZ) are seasonal increments seen under reflected light and black background. Specimen of 8 years old (33.1 cm FL) captured in Dzilam de Bravo in April 2008.

Right *sagittae* were embedded in clear epoxy and cut transversely at 300 µm thickness using a low-speed saw Isomet 1000 (Buehler). Digital black-and-white photos of the thin sections were taken, with chamomile oil to enhance contrast, using a stereomicroscope with an integrated camera (Leica EZ4E) attached to a PC. Age was determined based on the number of *annuli* counted with a stereomicroscope using reflected light and a black background [21]. In these conditions the seasonal increments or zones are visible as: the opaque zone (OZ) bright, and the translucent zone dark (TZ; Fig 2) [21]. Marginal increment analysis (MIA) was used to indirectly validate the annual deposition of one growth ring or *annulus* and the period: MIA =  (*R* - *r*_*i*_) / (*r*_*i*_ - *r*_*i-1*_) where *R* is otolith radius from center to edge, and *r*_*i*_ and *r*_*i-1*_ are the distances from the center to the last and penultimate OZ, respectively [22]. All distances were automatically measured using Age & Shape software (v. 10 Infaimon). Marginal increase was analyzed by *annuli* count and fishing site, accounting for changes in ring deposition period in response to specimen age and environmental conditions [23].

Following recommendations for obtaining age in Lutjanidae, otolith *annuli* were counted on the otolith´s ventral side, from the center to the edge, along an axis at the crest of the *sulcus acusticus* [23]. With no prior knowledge of individual size or fishing site, counts were done by two independent readers, in addition to the automatic count (Age & Shape v. 10, Infaimon). If readings did not match, the *annuli* were counted again to reach a consensus, but if discrepancies remained, the sample was rejected. Reading precision was estimated based on the general average percentage error (APE), the APE between readers, the APE of the automatic count (Age & Shape) and the general coefficient of variation (CV) [21,24,25].

Biological age or fractional age was calculated on an annual basis to estimate growth, natural mortality (*M*) and age of maturity. The calculation was done by adding the number of *annuli* to the fraction of time elapsed between theoretical date of birth and capture date [26]. Theoretical birth date corresponds to the average date, or the sum of days in the spawning season divided by two [27]; for *O. chrysurus* in the southern GoM this would be May 1^st^ [4]. For those individuals captured during the *annulus* deposition period but without an OZ at the otolith edge, one year was added on the assumption that a new OZ would deposit soon [28].

Growth curves by sex and fishing site were described using the von Bertalanffy growth model (VBGM): $E [L |t]={\text{L}}_{\infty }\left(1-{\text{e}}^{-\text{K}\left(\text{t }-{\text{t}}_{0}\right)}\right)$ where *E*[*L* | *t*] is mean length at age and L_ ∞ ,_ K and t_0_ are the parameters to be estimated [29]. The latter were interpreted, following [30], as L_ ∞ _: maximum mean length (FL, cm), K: speed at which the function attains L_ ∞_ (year^-1^), and t_0_ as the x-intercept. The VBGM was fitted using nonlinear least-squares estimations, and parameter 95% confidence intervals (CI) built by bootstrapping with 9999 iterations [30]. The growth curves were compared to determine if sex and fishing sites were statistically different. Given the number of parameters in the VBGM, the comparison between groups requires the fitting of up to eight growth curves. With the exception of one curve, all the remaining represent a discrepancy between groups (sex and fishing sites) for at least one of the parameters: one curve, the most “complex”, evaluates if all three parameters differ among groups (6 and 9 parameters for sexes and fishing sites, respectively). Three curves evaluate differences between two parameters (L_ ∞_ and K, L_ ∞_ and t_0_, and K and t_0_); three others evaluate differences in just one parameter (L_ ∞ _, K or t_0_); and the last one, the “coincident” curve, does not consider differences between the parameters among groups (3 parameters). The analysis was run hierarchically from the most complex to the coincident curve through likelihood ratio chi-squared test, according to the procedure described by [31]. The estimation of growth functions by group and the subsequent statistical comparisons were conducted within the R environment using the FSA package (v. 0.8) [32,33].

In order to facilitate the comparison of VBGM parameters with those from other studies conducted in different geographic areas, growth performance was estimated as follows: Ф’ = log(K) +  2 log (L∞), where, for the same species tends to be normally distributed [34]. A one-way ANOVA was used to identify differences in growth performance values between areas. The lifespan was calculated as *t*_*max*_ =  3/K +  t_0_ where K and t_0_ are the parameters defined with the VBGM [35].

Age at maturity (A_50_) by sex was calculated using generalized linear models with a binomial logit function with the *glm* function in R environment [32]. A bootstrap with 999 replicates was used to estimate the 95% CI with the plot maturity function in the *ggFishPlots* package [36,37]. Natural mortality (*M*) was calculated following [38]: *M* =  4.899*t*_*max*_^-0.916^. Age-based *M* values were estimated based on the VBGM: ln (*M*) =  0.55–1.61 ln (FL) +  1.44 ln (L_∞_) +  ln (K), where FL is fork length at the estimated age [39].

### Otolith shape

Whole left *sagittae* were photographed digitally in grayscale on a black background using a camera attached to a stereomicroscope (Leica EZ4E), and the LAS EZ software (Leica). Two images were taken of each, one with the *sulcus acusticus* facing up and the other facing down. All images were processed with Photoshop CS6 (v. 13.0, Adobe) to reduce visual imperfections [40]. Otolith variables were extracted by traditional morphometry from the digital images using Image Pro-Plus (v. 7.0, Media Cybernetics Software): 9 morphometric variables and 7 indices to describe otolith shape, and 6 morphometric variables and 2 indices to describe the *sulcus acusticus* (Table 1) [13,41]. The effects of fish size magnitude on otolith variables were eliminated using an ANCOVA and a homogeneity of slopes (*b*) test [42]. Variables that correlated with fishing site (main factor) and individual FL (covariate) were eliminated from the analysis. Variables that correlated only with FL were adjusted using the common in-group value of *b* [43,44].

**Table 1:** Description of the otolith (*sagittae*) and *sulcus acusticus* through morphometrics and shape indexes of *Ocyurus chrysurus.*

Otolith	Description
**Morphometrics**	
Area (A) *	Otolith area
Maximum diameter (MaxD) *	Length of the longest line joining two outline points and passing through the centroid
Minimum diameter (MinD)^§^	Length of the shortest line joining two outline points and passing through the centroid
Perimeter (P)^†^	Length of the otolith outline using a polygonal outline
*Rostrum* length (Rl)^†^	Length of the protuberance in the anterior margin usually including the anterior most point of the otolith
*Rostrum* width (Rw)^§^	Width of the protuberance in the anterior margin usually including the anterior most point of the otolith
*Antirostrum* length (Al)^†^	Length of the protuberance in the anterior margin, usually smaller than the *rostrum*
*Antirostrum* width (Aw)^†^	Width of the protuberance in the anterior margin, usually smaller than the *rostrum*
*Excisura* area (EA)^§^	Area of the portion of the anterior margin of the otolith where the *ostium* opens
**Shape indexes**	
Rectangularity (RE)^†^	Ratio between A/area of an imaginary bounding box around the otolith
Roundness (R) *	(P)2/(4π·A)
Ellipse (E) *	Perimeter of the ellipse surrounding the otolith outline
Fractal dimension index (Fi) *	(2·logP)/(logA)
Aspect (As)^†^	Ratio between major axis/minor axis of the ellipse. Always ≥ 1
*Rostrum* aspect (RAs)^†^	Ratio between *rostrum* width/ *rostrum* length
*Antirostrum* aspect (AAs)^†^	Ratio between *antirostrum* width/ *antirostrum* length
***Sulcus acusticus***	**Description**
**Morphometrics**	
*Sulcus* length (SAl)^§^	Length of the otolith internal face depression composed by the *ostium* and the *cauda*
*Sulcus* area (SAA)	Area of the otolith internal face depression composed by the *ostium* and the *cauda*
*Ostium* length (Osl) *	Length of the *ostium*
*Ostium* area (OsA)^§^	Area of the *ostium*
*Cauda* length (Cl)^§^	Length of the *cauda*
*Cauda* area (CA)	Area of the *cauda*
**Indexes**	
Percentage of the SAA occupied by the OsA^†^	*Ostium* area/ *Sulcus* area
Percentage of the SAl occupied by Osl	*Ostium* length/ *Sulcus acusticus* length

*Otolith variables corrected with the corresponding common in-group value of *b*.

§ Otolith variables removed because there was a positive correlation between FL and fishing sites.

† Otolith variables removed <  80% in the principal coordinates analysis.

Variation in otolith shape was estimated by analyzing the set of otolith variables, which were normalized to a common scale using the *scale* function. Indices expressed as percentages were transformed with the *arcsin* function before normalization. All statistical analyses were performed in R environment [45]. Once the variables were standardized, collinearity between variables was evaluated using the *corrplot* package [46], and a principal coordinates analysis (PCoA) done based on a Euclidean matrix and run with the *ape* package [47]. Based on the contribution values of axes one (PCoA 1) and two (PCoA 2), variables with a contribution percentage ≥  80% in either axis were selected [48]. To corroborate the influence of the factors fishing site, age and sex on otolith shape, a permutation-based multivariate analysis of variance (PERMANOVA) was applied (*p* =  0.001) [49]. Factors that influenced the variables were graphed using the *ggplot2* and *ggrepel* [50,51] packages. Finally, a homogeneity of variances test (betadisper) was done to identify any dissimilarity between groups and identify the group centroids, both using the *vegan* package [52].

## Results

### Age and growth

Size and weight of *O. chrysurus* from the southern GoM (n = 1,499) ranged from 16.3 – 41.8 cm (FL) and from 72.0 – 1,135.0 g (WW), respectively. A total of 88 otoliths were rejected because they were broken, and an additional 287 were either illegible or *annuli* readings did not coincide between readers. As a result, age and growth were quantified using 1,124 otoliths, of which 560 were from females (17.4 – 38.6 cm FL) and 564 from males (16.3 – 39.0 cm FL). The number of analyzed otoliths by fishing site was 338 from Celestún (mean ±  SD = 23.6 ±  2.25 cm FL), 391 from Dzilam de Bravo (mean ±  SD = 28.3 ±  3.81 cm FL), and 395 from Río Lagartos (mean ±  SD = 27.7 ±  3.47 cm FL). The largest female and male were caught at Dzilam de Bravo (38.6 and 39.0 cm FL, respectively), and the smallest female and male were caught at Celestún (17.4 and 16.3 cm FL, respectively) (Table 2; S1 Dataset).

**Table 2:** Data summary from 2008 and 2009 for *Ocyurus chrysurus* in southern Gulf of México.

Fishing site	n	Sex		FL (cm)	Mean	WW (g)	Mean	Sex		FL (cm)	Mean	WW (g)	Mean	Otoliths	
		Females			± S.D.		± S.D.	Males			± S.D.		± S.D.		
		I	Ma					I	Ma					Age	Shape
Celestún	528	22	243	17.4-25.0	23.2 ± 2.3	95.9- 290.4	213.7 ± 72.7	15	248	16.3-32.0	23.2 ± 2.3	72.0-615.6	213.0 ± 69.7	338	179
D. Bravo	449	0	232	21.1-36.0	29.0 ± 3.1	162.2- 847.7	433.0 ± 164.9	0	217	21.2-41.8	28.7 ± 3.4	162.6-1135.5	412.4 ± 164.9	391	158
R. Lagartos	472	0	229	21.6-38.3	28.1 ± 3.5	162.6- 496.1	386.4 ± 164.2	0	243	24.8-40.8	27.2 ± 3.4	152.3-967.6	344.9 ± 141.0	395	159
TOTAL	1,499	22	704	17.4-38.3		95.9- 847.7		15	708	16.3-41.8		72.0-1135.5		1,124	496

Number of individuals (n), minimum and maximum fork length in cm (FL), minimum and maximum whole-body weight in g (WW), individual sex and reproductive condition and number of otoliths used to calculate age and analyze shape. I = Immature, Ma = Mature, S.D. = standard deviation.

Reading accuracy was global APE = 3.19%, APE between readers =  4.0%, APE of the automatic count (Age & Shape) = 3.75% and CV = 4.64%. The growth rings were clear and well-defined, with concentric, alternating opaque and translucent zones. Beginning in the center of the otolith, these increased progressively towards the edge as individual size increased, the distance between zones diminishing with proximity to the edge (Fig 2).

**Fig 3:**
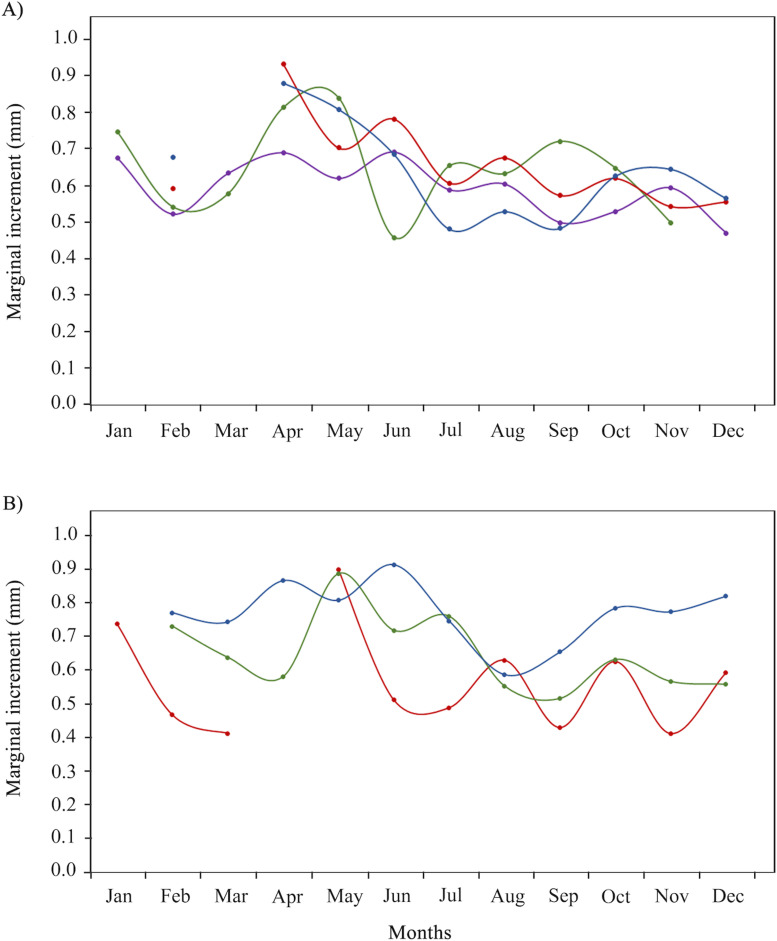
Marginal increment analysis of *Ocyurus chrysurus* in southern Gulf of México. A) By age: 3 years old (purple), 4 years old (green), 5 years old (red) and 6 years old(blue). B) By fishing site: Celestún (red), Dzilam de Bravo (green) and Río Lagartos (blue).

Overall, the deposition period – the period in which the shortest distance between the last *annulus* and otolith edge is recorded (i.e., marginal increment; MI) – occurred from July (*n* =  119, mean ±  SD: MI =  0.65 ±  0.40 mm) to September (*n* =  89, MI = 0.55 ±  0.32 mm). Differences in formation period of an *annulus* were observed when analyzing the MI per group age. Formation period in individuals with three *annuli* (*n* =  314) was July (MI =  0.59 ±  0.35 mm) to October (MI =  0.53 ±  0.32 mm); in those with four *annuli* (*n* =  138) it was June (MI =  0.46 ±  0.29 mm); in those with five *annuli* (*n* =  141) it was July (MI =  0.60 ±  0.36 mm) to November (MI =  0.54 ±  0.28 mm); and in those with six *annuli* (*n* =  80) it was July (MI =  0.48 ±  0.28 mm) to September (MI =  0.48 ±  0.39 mm; Fig 3A). This period of deposition of the *annulus* also varied between fishing sites: in Celestún it was February to March (*n* =  62, MI = 0.46 ±  0.30 mm; *n* =  23, MI = 0.48 ±  0.28 mm); in Dzilam de Bravo it was August to September (*n* =  34, MI = 0.54 ±  0.35 mm; *n* =  32, MI = 0.51 ±  0.25 mm); and in Río Lagartos from August to September (*n* =  37, MI = 0.58 ±  0.24 mm; *n* =  24, MI = 0.65 ±  0.40 mm; Fig 3B). Regardless of this variation in month of deposition, ring formation was confirmed to be annual.

Age ranged from 0^ +^ (young-of-the-year) to 12 years. The least frequent ages in both sexes were 0^ +^ (*n* = 10; FL = 17.75 ±  0.49 cm), 1 year (*n* = 15; FL = 19.35 ±  0.52 cm), and the older ages of 11 years (*n* = 23; FL = 35.89 ±  1.34 cm) and 12 years (*n* = 6; FL = 38.91 ±  0.95 cm). The most common ages were 2 and 3 years for females and for males (*n* = 160; FL = 23.0 ±  1.50 cm) (*n* = 133; FL = 25.6 ±  1.45 cm), (*n* = 170; FL = 22.90 ±  1.39 cm) (*n* = 155; FL = 25.5 ±  1.41 cm), respectively. Both sexes exhibited wide variability in size-at-age (Table 3A). This was particularly notable by fishing site. At Celestún, age ranged from 0^ +^ to 9 years and was the only site with young of the year (*n* = 9; FL =  17.6 ±  0.78 cm) and most of the one-year-olds (*n* = 13; FL =  19.0 ±  0.82 cm). The most infrequent ages in the site were 7 and 9 years (*n* = 1, FL =  31.4 cm; *n* = 2, FL =  32.2 ±  0.21 cm), and the most frequent was 2 years (*n* = 213; FL =  25.0 ±  1.22 cm), which represented 63.01% of the samples (Table 3B). At Dzilam de Bravo, ages ranged from 1 to 12 years, with 3 years (*n* = 82; FL = 26.0 ±  1.37 cm) and 5 years (*n* = 70; FL = 29.2 ±  1.08 cm) as the most frequent ages, and 1 year (*n* = 1; FL = 19.4 cm), 11 years (*n* = 11; FL = 37.0 ±  2.05 cm), and 12 years (*n* = 5; FL = 38.6 ±  0.21 cm) as the uncommon. The latter three ages accounted for only 4.34% of the samples from this site (Table 3B). At Río Lagartos, age ranged from 2 to 12 years, the most frequent age being 3 years (*n* = 125; FL = 25.70 cm ±  1.09), which accounted for 31.6% of the samples. The most infrequent ages were 10 years (*n* = 11; FL = 33.7 cm ±  0.73), 11 years (*n* = 14; FL = 36.2 cm ±  1.69) and 12 years (*n* = 2; FL = 41.0 cm), which, in combination, represent just 6.5% of the samples (Table 3B).

**Table 3:** Age-size keys of *Ocyurus chrysurus* from southern Gulf of México.

A)															
Females			Age (years)												
Ll	Ul	n	0^ + ^	1	2	3	4	5	6	7	8	9	10	11	12
16.00	18.18	5	4	0	1	0	0	0	0	0	0	0	0	0	0
18.18	20.36	11	2	6	3	0	0	0	0	0	0	0	0	0	0
20.36	22.55	54	1	1	50	2	0	0	0	0	0	0	0	0	0
22.55	24.73	117	1	0	96	20	0	0	0	0	0	0	0	0	0
24.73	26.91	92	0	0	9	76	7	0	0	0	0	0	0	0	0
26.91	29.09	97	0	0	1	34	37	25	0	0	0	0	0	0	0
29.09	31.27	106	0	0	0	1	1	42	45	17	0	0	0	0	0
31.27	33.45	50	0	0	0	0	0	1	4	5	8	29	3	0	0
33.45	35.64	15	0	0	0	0	0	0	0	0	0	3	8	4	0
35.64	37.82	9	0	0	0	0	0	0	0	0	0	0	0	9	0
37.82	40.00	4	0	0	0	0	0	0	0	0	0	0	0	1	3
40.00	42.18	0	0	0	0	0	0	0	0	0	0	0	0	0	0
	TOTAL	560	8	7	160	133	45	68	49	22	8	32	11	14	3
**Males**			**Age (years)**												
**Ll**	**Ul**	**n**	**0** ^** + **^	**1**	**2**	**3**	**4**	**5**	**6**	**7**	**8**	**9**	**10**	**11**	**12**
16.00	18.18	4	1	1	2	0	0	0	0	0	0	0	0	0	0
18.18	20.36	16	1	7	8	0	0	0	0	0	0	0	0	0	0
20.36	22.55	52	0	0	49	3	0	0	0	0	0	0	0	0	0
22.55	24.73	127	0	0	100	26	1	0	0	0	0	0	0	0	0
24.73	26.91	119	0	0	11	102	6	0	0	0	0	0	0	0	0
26.91	29.09	115	0	0	0	24	58	33	0	0	0	0	0	0	0
29.09	31.27	71	0	0	0	0	1	33	28	6	2	1	0	0	0
31.27	33.45	41	0	0	0	0	0	1	1	9	6	21	3	0	0
33.45	35.64	7	0	0	0	0	0	0	0	0	0	1	6	0	0
35.64	37.82	7	0	0	0	0	0	0	0	0	0	0	0	6	1
37.82	40.00	4	0	0	0	0	0	0	0	0	0	0	0	3	1
40.00	42.18	1	0	0	0	0	0	0	0	0	0	0	0	0	1
	TOTAL	564	2	8	170	155	66	67	29	15	8	23	9	9	3
**B)**															
**Celestún**			**Age (years)**												
**Ll**	**Ul**	**n**	**0** ^** + **^	**1**	**2**	**3**	**4**	**5**	**6**	**7**	**8**	**9**	**10**	**11**	**12**
16.00	18.18	9	7	2	0	0	0	0	0	0	0	0	0	0	0
18.18	20.36	29	2	11	16	0	0	0	0	0	0	0	0	0	0
20.36	22.55	76	0	0	70	6	0	0	0	0	0	0	0	0	0
22.55	24.73	151	0	0	118	33	0	0	0	0	0	0	0	0	0
24.73	26.91	41	0	0	9	29	3	0	0	0	0	0	0	0	0
26.91	29.09	24	0	0	0	8	13	3	0	0	0	0	0	0	0
29.09	31.27	5	0	0	0	0	1	1	3	0	0	0	0	0	0
31.27	33.45	3	0	0	0	0	0	0	0	1	0	2	0	0	0
33.45	35.64	0	0	0	0	0	0	0	0	0	0	0	0	0	0
35.64	37.82	0	0	0	0	0	0	0	0	0	0	0	0	0	0
37.82	40.00	0	0	0	0	0	0	0	0	0	0	0	0	0	0
40.00	42.18	0	0	0	0	0	0	0	0	0	0	0	0	0	0
	TOTAL	338	9	13	213	76	17	4	3	1	0	2	0	0	0
**Dzilam de Bravo**			**Age (years)**												
**Ll**	**Ul**	**n**	**0** ^** + **^	**1**	**2**	**3**	**4**	**5**	**6**	**7**	**8**	**9**	**10**	**11**	**12**
16.00	18.18	0	0	0	0	0	0	0	0	0	0	0	0	0	0
18.18	20.36	1	0	1	0	0	0	0	0	0	0	0	0	0	0
20.36	22.55	23	0	0	20	3	0	0	0	0	0	0	0	0	0
22.55	24.73	36	0	0	26	10	0	0	0	0	0	0	0	0	0
24.73	26.91	49	0	0	7	40	2	0	0	0	0	0	0	0	0
26.91	29.09	88	0	0	0	29	39	20	0	0	0	0	0	0	0
29.09	31.27	105	0	0	0	0	5	46	43	10	1	0	0	0	0
31.27	33.45	61	0	0	0	0	0	4	4	13	9	31	0	0	0
33.45	35.64	13	0	0	0	0	0	0	0	0	0	2	9	2	0
35.64	37.82	7	0	0	0	0	0	0	0	0	0	0	1	6	0
37.82	40.00	7	0	0	0	0	0	0	0	0	0	0	0	2	5
40.00	42.18	1	0	0	0	0	0	0	0	0	0	0	0	1	0
	TOTAL	391	0	1	53	82	46	70	47	23	10	33	10	11	5
**Río Lagartos**			**Age (years)**												
**Ll**	**Ul**	**n**	**0** ^** + **^	**1**	**2**	**3**	**4**	**5**	**6**	**7**	**8**	**9**	**10**	**11**	**12**
16.00	18.18	0	0	0	0	0	0	0	0	0	0	0	0	0	0
18.18	20.36	0	0	0	0	0	0	0	0	0	0	0	0	0	0
20.36	22.55	18	0	0	14	4	0	0	0	0	0	0	0	0	0
22.55	24.73	56	0	0	45	11	0	0	0	0	0	0	0	0	0
24.73	26.91	106	0	0	6	92	8	0	0	0	0	0	0	0	0
26.91	29.09	90	0	0	0	18	43	29	0	0	0	0	0	0	0
29.09	31.27	66	0	0	0	0	0	30	26	7	1	2	0	0	0
31.27	33.45	35	0	0	0	0	0	1	2	6	5	16	5	0	0
33.45	35.64	14	0	0	0	0	0	0	0	0	0	2	6	6	0
35.64	37.82	7	0	0	0	0	0	0	0	0	0	0	0	6	1
37.82	40.00	2	0	0	0	0	0	0	0	0	0	0	0	2	0
40.00	42.18	1	0	0	0	0	0	0	0	0	0	0	0	0	1
	TOTAL	395	0	0	65	125	51	60	28	13	6	20	11	14	2

Lower (Ll) and upper (Ul) limits in fork length (FL cm), and number of individuals per age (years). A) By sex: females and males, and B) By fishing sites: Celestún, Dzilam de Bravo and Río Lagartos.

**Fig 4:**
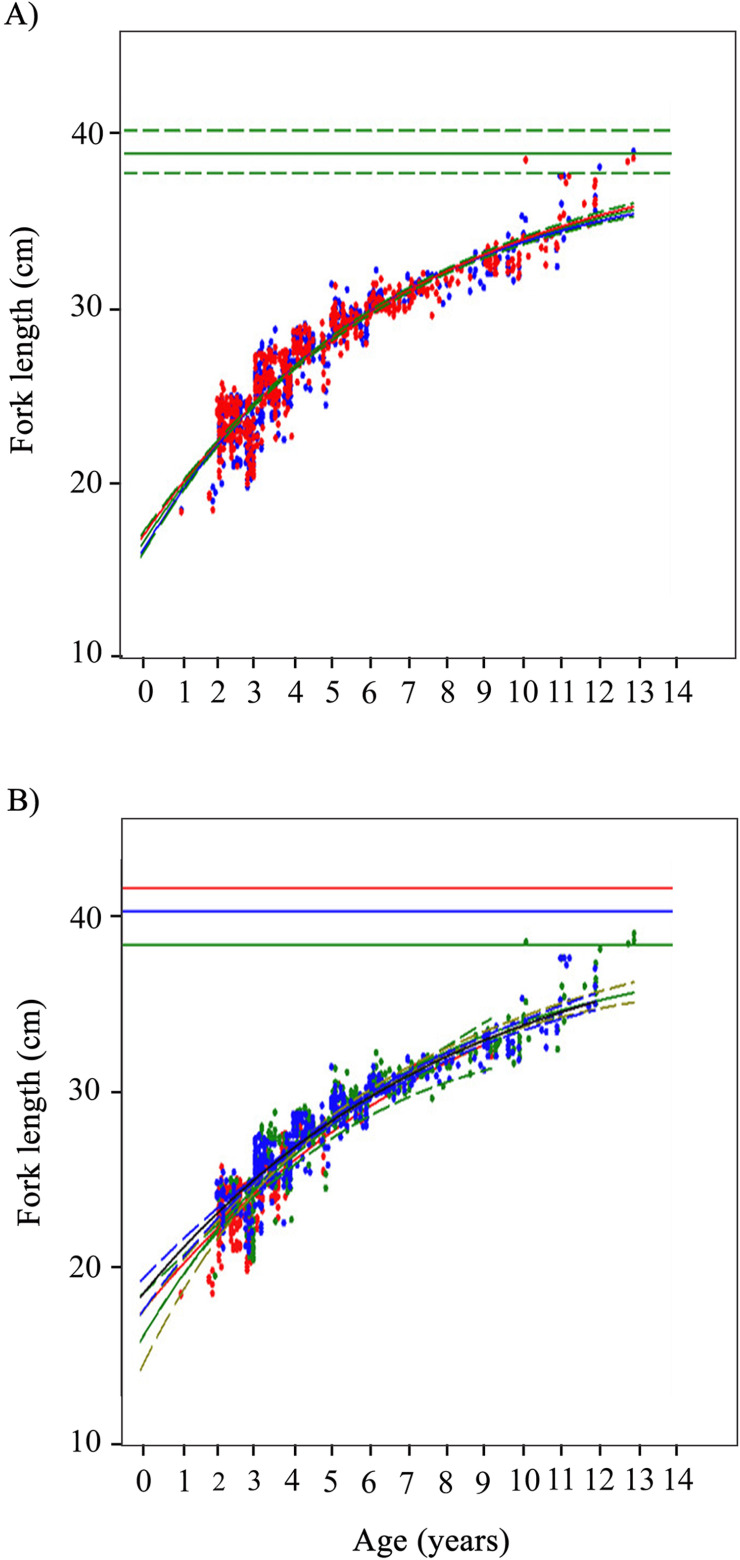
*Ocyurus chrysurus* von Bertalanffy growth curves calculated form biological age in years and fork length in cm. A) Growth curves for sex: both sexes (green), females (red), males (blue). Upper horizontal line corresponds to maximum mean length, whereas dotted lines correspond to 95% confidence intervals. B) Growth curves by fishing site: Celestún (red), Dzilam de Bravo (green) and Río Lagartos (blue). Upper horizontal lines correspond to maximum mean length per site, and the 95% confidence intervals are detailed in Table 4 to better appreciate the growth curve.

Growth estimates described through the VBGM for *O. chrysurus* were statistically significant (Table 4). The growth parameters and curves for females and males were very similar (Fig 4A), displaying no difference in the likelihood ratio when compared to the coincident curve with 3 parameters (*X*^2^ =  1.65, *P* =  0.65; Table 5). According to the growth estimates for both sexes, 95% of the L_ ∞_ value is reached around 15 years of age. Regarding to growth estimates by fishing sites Celestún and Río Lagartos showed the highest L_ ∞_ values and lowest K values, and Dzilam de Bravo the opposite (Table 4). The growth parameters estimated for Celestún presented a wide statistical variability. Specifically, the estimated value of L_ ∞_ (41.59 cm FL) and its upper confidence interval (59.72 cm FL) were 22% and 75% higher than the maximum size observed for the species at this site (34 cm FL; Fig 4B).

**Table 4:** Estimated von Bertalanffy growth parameters for *Ocyurus chrysurus* form southern Gulf of México by sex, with joined sexes (all) and by fishing sites.

	Parameter	S.E.	95% CI	*df*	*t*	*P*
**All**						
L_ ∞_ (cm)	38.85	0.62	37.77 – 40.19	1096	63.00	<2e-16 *
*K* (year ^-1^)	0.15	0.01	0.13 – 0.17		16.05	<2e-16 *
*t*_0_ (year^-1^)	-3.66	0.24	-4.16 – -3.22		-15.03	<2e-16 *
**Females**						
L_ ∞_ (cm)	39.58	0.96	36.95 – 41.78	542	41.37	<2e-16 *
*K* (year ^-1^)	0.14	0.01	0.12 – 0.16		10.94	<2e-16 *
*t*_0_ (year^-1^)	-3.97	0.37	-4.76 – -3.31		-10.82	<2e-16 *
**Males**						
L_ ∞_ (cm)	38.14	0.80	36.74 – 39.96	551	47.48	<2e-16 *
*K* (year ^-1^)	0.16	0.01	0.14 – 0.19		11.70	<2e-16 *
*t*_0_ (year^-1^)	-3.36	0.32	-4.03 – -2.78		-10.38	<2e-16 *
**Celestún**						
L_ ∞_ (cm)	41.59	5.95	34.37 – 59.72	316	6.99	1.63e-11 *
*K* (year ^-1^)	0.11	0.04	0.05 – 0.20		2.68	0.0078
*t*_0_ (year^-1^)	-4.86	1.15	-7.14 – -3.05		-4.21	3.28e-05 *
**Dzilam de Bravo**						
L_ ∞_ (cm)	38.36	0.93	36.80 – 40.65	380	41.46	<2e-16 *
*K* (year ^-1^)	0.16	0.02	0.13 – 0.20		9.04	<2e-16 *
*t*_0_ (year^-1^)	-3.25	0.48	-4.34 – -2.42		-6.83	3.42e-11 *
**Río Lagartos**						
L_ ∞_ (cm)	40.28	1.35	38.11 – 43.73	394	29.76	<2e-16 *
*K* (year ^-1^)	0.12	0.02	0.09 – 0.15		8.01	1.3e-14 *
*t*_0_ (year^-1^)	-4.99	0.57	-6.28 – -4.00		-8.71	<2e-16 *

L_ ∞_ =  mean maximum length, cm; FL, fork length; K = speed at which the function reaches L_ ∞ _, year^-1^; t_0_, x-intercept, year^-1^. S.E. = standard error of the parameter; CI =  confidence intervals at 95%; *df* =  degrees of freedom; *t* = t statistic value; *P* =  probability value. * Significant value (*P* <  0.001).

**Table 5:** Likelihood ratio Chi square test results of the comparison of von Bertalanffy growth curves among sex and fishing sites for *Ocyurus chrysurus.*

Test	m	*df*	LogL	*X* ^2^	*P*
^**ξ**^H_0_: L_ ∞ _, K, t_0_ no diﬀer among groups	3	1096	-1863.74		
H_0_ vs. H_1sex_:{L_ ∞ _, K, t_0_}	6	1093	-1862.91	1.65	0.647
H_0_ vs. H_1sites_:{L_ ∞ _, K, t_0_}	9	1090	-1840.59	46.30	2.582e-08 *
H_1sites_ vs. H_2sites_:{L_ ∞ _, K}, t_0_	7	1092	-1843.14	5.10	0.07806
H_1sites_ vs. H_3sites_:{L_ ∞ _, t_0_}, K	7		-1842.20	3.23	0.19927
H_1sites_ vs. H_4sites_:{K, t_0_}, L_ ∞ _	7		-1841.33	1.49	0.47562
H_4_ vs. H_5sites_:{K}, L_ ∞ _, t_0_	5	1094	-1845.93	9.20	0.01008 *
H_4_ vs. H_6sites_:{t_0_}, L_ ∞ _, K	5		-1845.91	9.15	0.01028 *
H_0_ vs. H_5sites_	5		-1845.93	35.62	1.844e-08 *
H_0_ vs. H_6sites_	5		-1845.91	35.66	1.807e-08 *

L_ ∞_ =  mean maximum length, cm; FL, fork length, cm; K = speed at which the function reaches L_ ∞ _, year^-1^; t_0_, x-intercept, year^-1^. The braces denote which parameters diﬀer among groups. ^**ξ**^Corresponds to the coincident curve that does not contemplate differences between growth parameters among groups. m =  numbers of parameters; *df* =  degrees of freedom; LogL =  Log-likelihood value; *X*^*2*^ =  Chi square value; *P* =  probability value. * Significant value (*p* <  0.05).

The likelihood ratio indicates substantial differences between the coincident curve (3 parameters) and the most complex curve (9 parameters) per fishing site (*X*^2^ =  46.3, *P* <  0.0001). In the subsequent step, the latter growth curve was selected for comparison with 7 parameters curves, which only consider a difference in two parameters per fishing site. The likelihood ratios of these three curves did not show significant differences at 95% confidence with the more complex one (*P* >  0.05), and so they are considered better than the more complex curve because they fit the observed data equally well but are more parsimonious. Of these three possible curves, the one that considers differences between K and t_0_ by fishing site presented the highest log-likelihood value (-1841.33), so it was selected for comparison with the lower hierarchical growth curves of 5 parameters. The results showed that both K and t_0_ differ between the three fishing sites (*P* values ≈  0.01). A comparison of these curves independently with the coincident curve confirmed that the difference was attributed to K and t_0_ (*P* values <  0.0001; Table 5).

**Fig 5:**
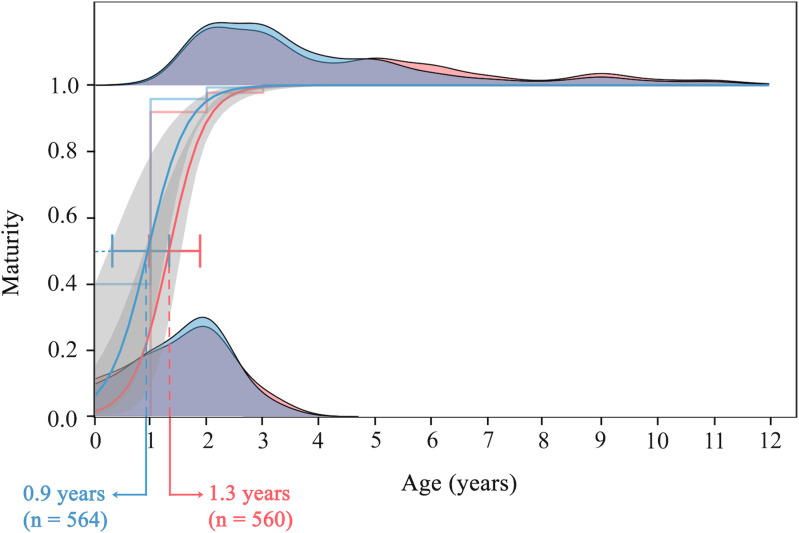
*Ocyurus chrysurus* maturity ogive by age in southern Gulf of México. Females (red) and males (blue) distributions in 0 correspond to inmature individuals, whereas distributions in 1 correspond to mature individuals (*n* =  1,124). Solid lines represent logistic general lineal function and grey shading their standard error. Dotted lines show age at maturity (A_50_) in years per sex. Horizontal error bars indicate confidence intervals of A_50_.

Age at maturity was 1.3 years for females and < 1 year for males (Fig 5). The youngest mature female was one year old (19.9 cm FL), and the youngest mature male was < 1 year (0^ + ^, 18.5 cm FL). All females were mature at 2 – 3 years, and males at 2 years. Due to the early sexual maturation of the species, there were not enough immature individuals to meaningfully analyze possible differences in age at maturity by fishing site, since, for example, in Río Lagartos, there were no individuals younger than 2 years old (all of the specimens of the site were mature).

**Fig 6:**
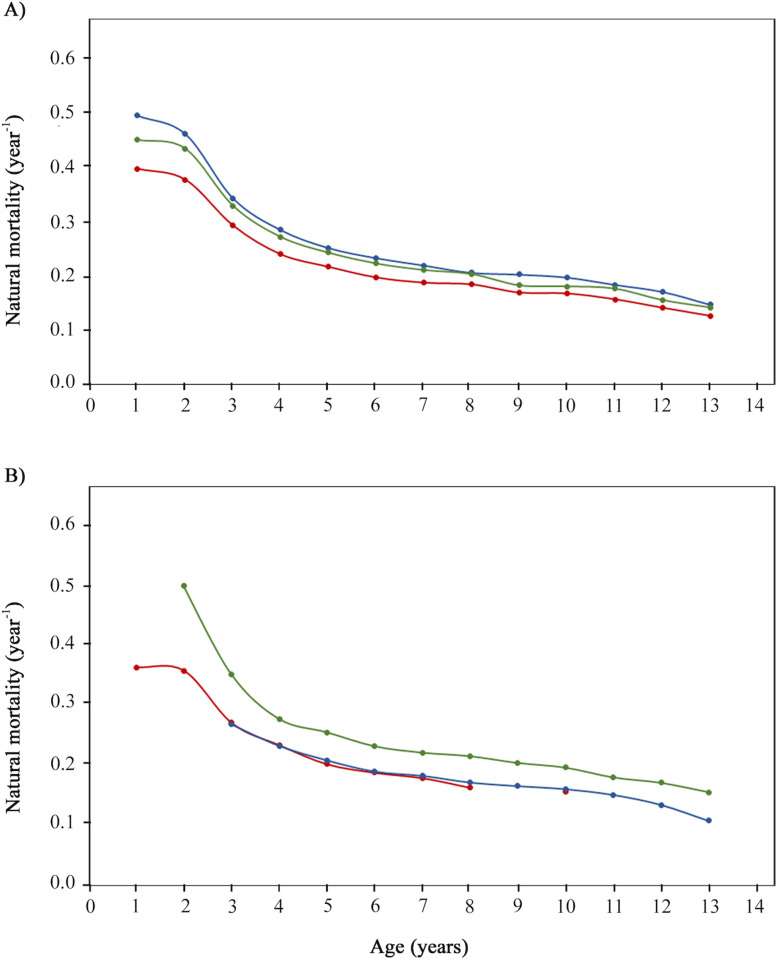
Natural mortality by age for *Ocyurus chrysurus* in southern Gulf of México. A) By age per sex: both sexes (green), females (red) and males (blue). B) By age per fishing site: Celestún (red), Dzilam de Bravo (green) and Río Lagartos (blue).

Natural mortality (*M*), calculated by sex and fishing site, decreased exponentially from the youngest to the oldest individuals. This parameter based on age was lower in females at all ages: females, 0.39– 0.12 year^-1^; males, 0.49 – 0.14 year^-1^ (Fig 6A). By fishing site, *M* at age was lower at Río Lagartos (0.26 – 0.10 year^-1^) than at Celestún (0.36 – 0.15 year^-1^) and Dzilam de Bravo (0.49 – 0.15 year^-1^). At all three sites, *M* at age was higher among young individuals than in older ones. At Celestún, it was 0.36 – 0.20 year^-1^ for the young (0^ +^ – 4 years) and 0.18 – 0.15 year^-1^ in adults (5 - 9 years). At Dzilam de Bravo it was 0.49 – 0.25 year^-1^ for the young (1 – 4 years) and 0.22 – 0.15 year^-1^ for adults (5 – 12 years). And at Río Lagartos, it was 0.26 – 0.21 year^-1^ for the young (2 – 4 years) and 0.19 – 0.10 year^-1^ for adults (5 – 12 years) (Fig 6B). Overall *M* values were 0.37 year^-1^ in the studied population, 0.32 year^-1^ for females, 0.40 year^-1^ for males, 0.28 year^-1^ for Celestún, 0.39 year^-1^ for Dzilam de Bravo, and 0.31 year^-1^ for Río Lagartos.

### Otolith Shape

Four otolith and four *sulcus* variables were eliminated from the analysis because they correlated with fishing site and FL: minimum diameter (MinD), *Rostrum* width (Rw), *Excisura* area (EA), *Antirostrum* aspect (AAs) and *sulcus acusticus*: *Sulcus* length (SAl), *Ostium* area (OsA), *Cauda* length (Cl), and percentage of SA1 occupied by Osl (Table 1). Seven otolith and s*ulcus* variables were corrected because they correlated only with FL (*b* value in parenthesis): Area (A, *b* = 1.4235), Maximum diameter (MaxD, *b* =  0.1767), Perimeter (P, *b* =  0.5842), Roundness (R, *b* =  0.0102), Ellipse (E, *b* =  0.4316), Fractal dimension index (Fi, *b* =  0.0008) and *Ostium* length (Osl, *b = * 0.0708; S2 Dataset).

**Fig 7:**
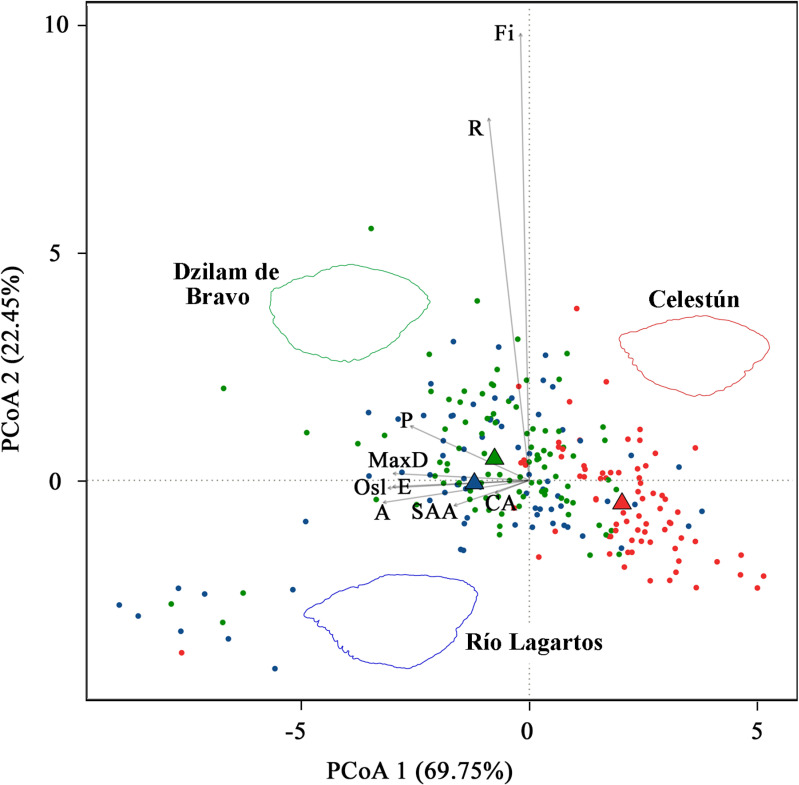
Principal coordinates analysis (PCoA) plot on the otolith shape of *Ocyurus chrysurus.* Otolith shape based on Euclidean distance matrix, depicting patterns of otolith morphometrics and shape indexes according to fishing sites: Celestún (red), Dzilam de Bravo (green) and Río Lagartos (blue). Triangles represent group centroid. Outlines of the otolith contour show 2-year-old individuals from each fishing site according to their color. R = Rectangularity, Fi = Fractal dimension index, P = Perimeter, MaxD = Maximum diameter, Osl = *Ostium* length, E = Ellipse, A = Area, SAA = *Sulcus acusticus* area, CA = *Cauda* area.

In the PCoA analysis, the variables that made a > 80% contribution in PCoA1 were A (95.33%), MaxD (94.85%), P (93.53%), E (95.45%), SAA (93.44%), Osl (88.525), and CA (90.035), while for PCoA2 they were R (87.17%) and Fi (81.85%). Using these variables in the PERMANOVA analysis showed that otolith shape differed between fishing sites and between individuals aged 2-, 3-, 6- or 9-years’ age. There were no differences between sexes observed in otolith shape (Table 6). Between fishing sites, at Dzilam de Bravo (*n* =  93) and Río Lagartos (*n* =  71) otolith shape was larger and wider, with a more developed s*ulcus acusticus*. Of note is that otoliths from Dzilam de Bravo were rounder with a smoother edge than those from Río Lagartos, which tended to be more elliptical with an irregular or fractal edge. In contrast, otoliths from Celestún (*n* =  77) were smaller, with a less developed *sulcus acusticus* (Fig 7). Those from Celestún were also lighter (*n* =  77, OW =  0.060 ±  0.034) than those from Dzilam de Bravo (*n* =  93, OW =  0.092 ±  0.034) and Río Lagartos (*n* =  71, OW =  0.111 ±  0.090). In the PCoA, otolith differentiation by fishing site was 69.75% on PCoA1 and 22.45% on PCoA2, meaning that otolith variables explained 92.2% of the separation between groups (Fig 7). In the betadisper analysis, average distance of the centroids for each group by fishing site was 1.98 for Celestún, 2.22 for Dzilam de Bravo, and 2.75 for Río Lagartos (Fig 7). Otolith shape variations in response to age was analyzed using only those from 2-year-old individuals since this age group was the largest at each site, confirming that otolith shape differed between those from Celestún (*n* =  20) and those from Dzilam de Bravo (*n* =  19)/ Río Lagartos (*n* =  12; Table 6).

**Table 6:** PERMANOVA results testing for differences in otolith shape of *Ocyurus chrysurus* between three fishing sites (Celestún, Dzilam de Bravo y Río Lagartos), sex (females and males) and ages (1 year – 12 years old) and 2 years old.

Source of variation	*df*	SS	*n*	F-exp	*p*-value
**Fishing sites**					
Celestún	1	339.18	238	49.27	0.0009 *
Dzilam de Bravo	1	76.53	238	11.11	0.0009 *
Río Lagartos	1	105.93	238	15.38	0.0009 *
Total	2	521.64	238	37.88	0.0009 *
**Sex**					
Females	1	8.84	239	0.98	0.1188 *
Males	1	9.84	239	1.09	0.1188 *
Total	1	18.69	239	2.08	0.1188 *
**Age**					
1	1	4.02	229	0.55	0.5034 *
2	1	84.12	229	11.63	0.0009 *
3	1	101.69	229	14.06	0.0009 *
4	1	1.69	229	0.23	0.7772 *
5	1	36.98	229	5.11	0.0049 *
6	1	94.33	229	13.04	0.0009 *
7	1	13.32	229	1.84	0.1258 *
8	1	7.55	229	1.04	0.2717 *
9	1	68.76	229	9.50	0.0019 *
10	1	64.67	229	8.94	0.0039 *
11	1	6.28	229	0.86	0.2977 *
12	1	20.16	229	2.78	0.0649 *
Total	11	503.63	229	6.32	0.0009 *
**2 years old**					
Celestún	1	46.27	48	6.75	0.0009 *
Dzilam de Bravo	1	69.66	48	10.17	0.0009 *
Río Lagartos	1	5.28	48	0.77	0.3246 *
Total	2	121.23	48	8.84	0.0009 *

*df* =  degrees of freedom, SS =  Sum of squares, *n* =  number of individuals, F–exp = F value by permutation, *p*-value = based on 1000 permutations.

*Significant value (*p* < 0.0001).

## Discussion

### Age and growth

The size of *O. chrysurus* captured in the southern GoM ranged in size from 16.3 – 41.8 cm FL (mean ±  SD = 25.1 ±  3.9 cm), which is comparable to the size of individuals caught by the artisanal fishing fleet of Yucatán. This size range is indicative of the selectivity of the main fishing gears used for this resource. Although hand lines, longlines and spear diving are the main gears used by artisanal finfish fishery in the State, approximately 99% of *O. chrysurus* capture is done with hand line and longline using circular hooks sizes #7 to #10 [8]. The reported size range of 18 – 46 cm FL (mean ±  SD = 26.6 ±  3.2 cm) for *Ocyurus chrysurus* specimens captured by the artisanal fleet [8], is very similar to the size range reported in this study. The smallest individuals were captured in the western part of the Yucatán Peninsula, towards Celestún, whereas the largest were captured towards the east, in the area of Río Lagartos, which also coincides with the results of this study. Individuals exceeding 46 cm (FL) are seldom encountered in Yucatán, yet individuals up to 60 cm TL have been documented in the central GoM in the state of Veracruz, captured using hooks #3 [53,54]. Consequently, variations in size can be attributed to the selectivity of the fishing gear employed in specific GoM fishing areas, associated with hook characteristics [55]. The largest size recorded for *O. chrysurus* was 75 cm TL in the Bahamas and 86.3 cm TL in Venezuela [56,57].

*Annuli* formation period in the studied otoliths varied by age and fishing site, a phenomenon observed in other fish species and other snappers such as *L. campechanus* [25,58]. Considering this, we followed [23] recommendations for analyzing marginal increment in Lutjanids: do ring counts randomly without knowledge of catch date, analyze age groups separately, and use samples collected from at least two years. Unfortunately, most studies of the *annuli* formation period in *O. chrysurus* report the overall period. To allow comparison, overall formation period was calculated in the present results, which is July to September, although when broken down by age group it spreads from June to November. In either case, the period in the present results is longer than reported elsewhere: March and May for individuals aged 1 to 6 years in the Virgin Islands and Puerto Rico [59]; April to June in southern Florida [60,61]; March to June on the Atlantic coast of the USA [62]; February to May in the south Atlantic of the USA [63]; April to May in Cuba [64]; and May to August in Brazil [65]. The period of *annuli* formation in the southern GoM is extensive, as has been seen in other snapper species with extended spawning seasons, e.g., *L. campechanus, L. synagris* and *Lutjanus griseus* (Linnaeus 1758, Gray snapper) [66]. For *O. chrysurus* in southern GoM, the formation of the *annuli* occurs during their reproductive period (January – September), specifically after the May and September spawning peaks [4].

Age ranged from 0^ +^ to 12 years, the highest recorded age for this species in the southern GoM, with no differences between sexes. The number of individuals with 10 –12 years’ age in the present study is higher (n = 49; FL =  31.2 – 40.0 cm) than reported in other studies of *O. chrysurus*. Twelve individuals (FL =  35.5 – 50.5 cm) older than 10 years were reported in the Virgin Islands and Puerto Rico [59]; only two aged 12 and 14 years (FL =  46.8 cm and 44.3 cm) were reported in the South Atlantic in Florida [60]; three aged 10 to 13 years (50.0 – 55.0 cm FL) were reported in the northeast GoM [62]; and 3.2% of the sample was older than 10 years in southern Florida [63]. The oldest reported age for *O. chrysurus* is 23 years in Florida and the GoM, and 19 years in Brazil [65,67]. In contrast, individuals aged 2 to 4 years are the most abundant in age and growth studies of *O. chrysurus*. In the present results, these age groups represented 65% of the sample, and in other populations close to the 70% - 88% are reported in these age groups [60,62,63]. These values are consistent even in samples collected in fishery-independent contexts [68].

Size-at-age in *O. chrysurus* varies widely in this and other studies, regardless of sex. For instance, individuals from 2 to 4 years of age in the present results display 18.2 – 31.3 cm FL. This is comparable to the size range reported elsewhere for the same age group: 12.4 – 46.2 cm FL [60]; 18.4 – 26.4 cm FL [59]; 25.3 – 35.8 cm FL [64]; 22.5 – 42.4 cm FL [62]; and 22.5 – 60.0 cm FL [68]. Clearly, length is not an accurate predictor of age in *O. chrysurus* and therefore estimating age via length frequencies is not recommended [61–63,68].

A common issue in fisheries is the paucity of age-length data from young or old fish. In the absence of data form young individuals, the representation of the “early” part of the VBGM curve, will be compromised [69]. Similarly, without older fish it may be difficult to characterize the asymptote of the VBGM curve [69]. Growth estimation is of particular importance for population dynamics and fishery management, since species with faster growth rates tend to support higher exploitation rates, than slow-growth species [70]. Growth misspecification can affect the age structure of the population and thus can bias natural and fishing mortality, biomass estimates and the associated reference points and stock status [71,72]. Our growth estimates may be biased based on the underrepresentation of younger individuals ( < 2 years), as evidenced by the low t_0_ values obtained. When size-at-age data is analyzed, it is important to consider the nature of the sample and in particular, how it may have been affected by gear selectivity (based on size or age) [73]. Therefore, since fishing gear has an important effect on size-based selectivity for *O. chrysurus*, a fishery-independent sampling is necessary in future research.

Variations in size-at-age have also been observed between north and south Florida *O. chrysurus’* populations, between time periods and distribution areas [59,60,62,63]. In all cases, intense fishing pressure plays a role in age and size variations. Fishing records show that Celestún is the home port for the largest number of vessels in Yucatan’s artisanal fishing fleet (n =  749 vessels), and, perhaps not coincidentally, is the fishing site where the smallest *O. chrysurus* are landed [8]. Apparently, fishing pressure at Celestún is greater than at Dzilam de Bravo or Río Lagartos, both of which have *O. chrysurus* of wider size and age distribution. Overexploited stocks typically exhibit a truncated length and age distribution due to the continuous extraction of large individuals [74,75].

The estimated VBGM parameters also differ between fishing sites, but the composition of age and size-at-age data should be considered. Celestún had the youngest individuals (young-of-the-year to 9 years), as well as the smallest (16.0 - 34.0 cm FL). At Río Lagartos, age ranged from 2 to 12 years, and sizes were the largest of the three sites (19.0 – 43.0 cm FL). As mentioned above, without older fish the L_ ∞_ might be overestimated and with a very low estimate of K (possible scenario for Celestún). On the contrary, without younger individuals the t_0_ might be much lower and thus affect growth rate (possible scenario for Río Lagartos). Therefore, caution should be made if the growth parameters for these two fishing sites are to be used in stock assessments.

Reported growth parameters for *O. chrysurus* vary widely between distribution areas (Table 7). Values for L_ ∞_ vary from 30.1 to 68.0 cm FL; the highest values are generally reported in the Caribbean and southern Florida. Estimates of K vary from 0.13 to 0.5 year^-1^, although there is no clear relationship to geographic area since most values are < 0.2 year^-1^, characteristic of moderate-growing species. The Ф’ values ranged from 2.3 to 3.1. The lowest values were those obtained in this study. However, the index was normally distributed around a mean of 2.67 (W =  0.971, P =  0.67), which coincides with that reported by [64] based on 13 growth studies. No evidence was found to support differential growth between geographic areas (F_2,22_ =  2.31, P =  0.12).

**Table 7:** Growth parameters for *Ocyurus chrysurus* estimated in several distribution areas.

L_ ∞ _	K	t_0_	Ф’	*t*_max_	A_max_	Sex	Region	Method	Reference
45.1	0.3	-0.4	2.7	10.8	14	A	S Florida	O	[60]
30.1	0.5	-1.7	2.6	4.7	–	A	SE Florida	O	[68]
61.8	0.1	-3.1	2.7	19.9	–	A	Florida	O	[67]
48.4	0.2	-1.9	2.6	15.8	–	A	SE US	O	[62]
44.6	0.5	-0.6	3.0	5.1	–	A	SE US	O	[61]
41.0	0.3	-2.0	2.7	9.1	–	A	S US Atlantic	O	[63]
53.0	0.1	–	2.4	30.0	10	A	S GoM	S	[76]
45.3	0.2	–	2.5	18.8	–	A	S GoM	S/ L-f	[77]
39.5	0.1	-4.0	2.3	17.5	12	F	S GoM	O	This study
38.1	0.2	-3.4	2.4	15.4	12	M	S GoM	O	This study
38.9	0.2	-3.7	2.4	16.3	12	A	S GoM	O	This study
51.0	0.5	–	3.1	6.1	7	A	S GoM	S	[54]
49.2	0.3	-1.2	2.8	10.3	8	A	NW Cuba	V/ SC	[78]
50.0	0.2	-0.8	2.6	19.2	–	A	Cuba	V/ SC	[79]
68.0	0.2	-0.9	2.9	18.0	6	A	SW Cuba	O	[64]
60.4	0.2	-0.7	2.9	14.3	–	M	SW Cuba	O	[64]
55.6	0.2	-0.5	2.9	12.0	–	F	SW Cuba	O	[64]
48.9	0.3	-0.3	2.9	9.4	–	F	NW Cuba	O	[64]
47.7	0.3	-0.2	2.9	9.1	4	M	NW Cuba	O	[64]
69.9	0.1	-0.9	2.7	29.2	6	A	SE Cuba	UB	[80]
61.6	0.2	-0.7	2.8	19.4	7	A	Cuba	O	[81]
60.0	0.25	–	2.9	12.0	–		Jamaica	L-f	[56]
50.2	0.1	-1.0	2.5	20.6	17	A	Virgin Is./PR	O	[59]
49.5	0.1	-2.5	2.4	25.5	–	A	Brazil	O	[82]
56.7	0.1	-0.8	2.6	22.3	–	A	Brazil	O	[65]

L_ ∞_ =  mean maximum length in fork length, cm; K = speed at which the function reaches L_ ∞ _, year^-1^; t_0_, x-intercept, year^-1^; Ф’ =  growth performance index; *t*_*max*_ =  life span, years; A_max_ =  maximum registered age, years; Sex: A =  all sexes pooled; F =  females; M =  males; Method: O =  otoliths; S =  scale; V =  vertebra; SC =  supraoccipital crest; UB =  urohyal bone; L-f =  length frequency. Values for Ф’ and *t*_*max*_ were calculated from previous studies to compare

Reported species lifespan varies from 4.7 years in Florida [68] to 22.3 years in Brazil [65], regardless of sex (Table 7). In the US Atlantic and northern GoM populations, lifespan varies from 4 to 19 years, while in Caribbean populations it fluctuates from 9 to 22 years [83]. In the present results, lifespan in females from the southern GoM was 19.1 years but 15.3 years in males. Differences in lifespan by sex have also been recorded in Cuban populations (12.2 years for females vs. 14.2 years for males) [64]. Lifespan may varied between populations due to differences in environmental and genetic factors or food availability that influence growth rate. Since lifespan is a *K*-based parameter, these differences were expected.

Age at maturity has not been reported extensively for *O. chrysurus*. Otolith analysis of a Florida population showed A_50_ =  1.7 years [67], whereas scale analysis in a population in Veracruz, México found A_50_ =  2 years [53], otolith analysis of a population in Cuba found A_50_ =  2 years [84], and the present results from the southern GoM were A_50_ =  1.3 years for females and < 1 year for males. The rapid changes in size-at-age, may reflect the inability of an overfished or depress population to absorb or respond to further decreases in population size [85]. If fishing pressure eliminates individuals genetically predisposed towards rapid growth and larger sizes, and only left the remaining individuals that tend to grow more slowly and attain smaller sizes [85], it might be the explanation of the absence of older and larger individuals in Celestún, since as exposed previously, this site has much more fishing pressure than the other two sites. Nevertheless, further studies are required on the Celestún’s population in order to confirm this demographic change.

Data on overall natural mortality (*M*) in *O. chrysurus* are limited. Reported values range from 0.19 – 0.40 year^-1^, with the lowest value (0.19 year^-1^) in Florida [67], and the highest in Cuba and the southeast US (0.40 year^-1^) [61,81], and the southern GoM (0.37 year^-1^, present results). Natural mortality at age gradually decreases from the youngest (0^ +^ to 3 years) to 10 + years, when mortality remains near 0.1 year^-1^. This is characteristic of tropical fishes in which predation of juveniles is the main factor modeling *M* [39].

### Otolith Shape

Otolith shape responds to genetic, biological and ecological factors [44,86,87]. In *Lutjanus* species, this parameter exhibits high intra- and interspecies variability derived mainly from the influences of sex, age, environmental and genetic characteristics [88–91]. Environmental factors such as salinity, water temperature and depth are responsible for morphological differences such as *sulcus acusticus* area and otolith size [92,93].

In *O. chrysurus*, otolith shape differs between fishing sites, possibly due to environmental variations. Exogenous factors are known to directly influence shape indices, while endogenous factors affect morphometric variables [94,95]. In the present results, the shape indices that distinguished between groups by site were fractal dimension index, roundness and ellipse, suggesting that some of the observed differences respond to specific environmental factors found at each site. Water temperature is also known to affect fish growth and therefore otolith shape [88]. For example, average spring-summer bottom temperature at Río Lagartos was 20.5 ºC but 22.9 ºC at Celestún, a 2 + ºC difference caused by upwelling of cold, nutrient-enriched waters at Río Lagartos [16,96]. Of the three sites, Celestún had both the highest temperature and the highest salinity [97,98], which also affects otolith shape [93]. Río Lagartos has the hardest and most complex bottoms, and otoliths from this site were more elliptical in shape with irregular edges, which agrees with previous reports [86].

Environmental conditions influence growth rate, thereby affecting otolith morphology. For instance, individuals exhibiting accelerated growth patterns typically possess otoliths that are characterized by increased length and reduced weight [88,99]. In the present results, individuals from Celestún had the smallest growth rate (K) but the lightest otoliths. This could be due to the generally younger age of the Celestún sample (i.e., younger individuals have lighter otoliths than those of older individuals), but the analysis of otolith shape in two-year-old individuals shows clear differences in shape remain between Celestún and Dzilam de Bravo/Río Lagartos. The different conservation conditions at each site may also have affected this parameter. Celestún and Río Lagartos are federal-level Biosphere Reserves since both have lagoon systems harboring protected species; however, neither includes a marine protection zone. At Dzilam de Bravo, the Dzilam State Reserve (DSR) includes a 17,513-ha marine protection strip corresponding to 33% of the protected area [100]. Due to the DSR, some fishers have shifted from fishing to ecotourism, decreasing fishing pressure at this site [101].

Trophodynamics may also affect growth, and thus otolith shape, at the three studied fishing sites. In a previous study about diet composition for the same individuals analyzed in this study [3], Penaeioidea shrimp was the most common prey in the stomach contents of *O. chrysurus* at all three sites, although Caridea crabs were highly frequent at Celestún, and Osteichthyes fish at Dzilam de Bravo [3]. No Osteichthyes and very few Caridea were documented at Río Lagartos [3]. Differences in prey biomass and composition were recorded between the three sites in the stomach contents analyzed [3]. Otolith shape is directly linked to fish diet, in that different prey categories produce broad and fine variations in the contour, with the relative diet composition contributing more to this variability than the quantity of prey ingested [102,103]. The predominance of Penaeioidea shrimp in the diet of *O. chrysurus* at Río Lagartos [3] could be possible related to the more complex otoliths (higher fractal dimension index) observed from this site.

Otolith shape may change in response to ontogenetic migrations from settlement areas to juvenile breeding areas and then to adult areas for reproductive aggregations [2]. However, we think that these movements probably occur in different zones but within the same fishing site. A study of *O. chrysurus* in the Florida Keys using acoustic tagging showed individuals to have very restricted movements, with approximately 60% of marked individuals exhibiting high site fidelity [104]. High post-settlement site fidelity has also been confirmed for populations of *Ocyurus chrysurus* from the British Virgin Islands [105].

Growth characteristics are an integral component of fish stock assessments. By relating size to age, growth modeling can be useful to estimate life history parameters such as growth rate, maximum mean length, longevity, and natural mortality. According to our results, *O. chrysurus* is characterized by medium size, moderate-growth, early age-at-maturity and medium lifespan. The observed inter-site differences in otolith shape suggest that the southern GoM *O. chrysurus* population has structural complexities not previously established for this species in this region. The incorporation of demographic data into harvest strategies can facilitate sustainable fishery management [106]. Developing a management plan for *O. chrysurus* in the southern GoM that considers its population structure is urgent due to constantly increasing catch volumes and the demographic changes in process at the studied fishing sites.

## Supporting information

S1 Data Data set of *Ocyurus chrysurus* with fishing site, age, length, sexual maturity, and sex variables used to obtained growth parameters, age at maturity, lifespan, and natural mortality. (XLSX)

S2 Data Data set of *Ocyurus chrysurus* with otolith and *sulcus acusticus* morphometrics and indices used to analyze the otolith shape by fishing site. (XLSX)
